# Hip-preserving reconstruction using a customized cemented femoral endoprosthesis with a curved stem in patients with short proximal femur segments: Mid-term follow-up outcomes

**DOI:** 10.3389/fsurg.2022.991168

**Published:** 2022-09-22

**Authors:** Qi You, Minxun Lu, Li Min, Yuqi Zhang, Yi Luo, Yong Zhou, Chongqi Tu

**Affiliations:** ^1^ Department of Orthopedics, Orthopedic Research Institute, West China Hospital, Sichuan University, Chengdu, China; ^2^ Sichuan Model Worker and Craftsman Talent Innovation Resaerch Studio, China

**Keywords:** customized cemented femoral endoprosthesis, intra-neck curved stem, massive femoral diaphyseal defects, short proximal femur segment, reconstructive surgery

## Abstract

**Background:**

Short metaphyseal segments that remain following extensive distal femoral tumor resection can be challenging to manage, as the residual short segments may not be sufficient to accept an intramedullary cemented stem of standard length. The present study was developed to detail preliminary findings and experiences associated with an intra-neck curved stem (INCS) reconstructive approach, with a particular focus on mechanical stability.

**Method:**

From March 2013 to August 2016, 11 total patients underwent reconstructive procedures using a customized cemented femoral endoprosthesis (CCFE) with an INCS. Measurements of femoral neck-shaft angle values were made before and after this procedure. Radiological outcomes associated with this treatment strategy over an average 63-month follow-up period were additionally assessed. Functionality was assessed based upon Musculoskeletal Tumor Society (MSTS) scores, while a visual analog scale (VAS) was used to rate pre- and postoperative pain, and any complications were noted.

**Results:**

Relative to the preoperative design, no significant differences in femoral neck–shaft angle were observed after this reconstructive procedure (*p* = 0.410). Postoperatively, the tip of the stem was primarily positioned within the middle third of the femoral head in both lateral and posterior-anterior radiographic, supporting the accuracy of INCS positioning. The average MSTS score for these patients was 25 (range: 21–28), and VAS scores were significantly reduced after surgery (*p* < 0.0001). One patient exhibited local disease recurrence and ultimately succumbed to lung metastases, while two patients exhibited aseptic loosening. None of the treated patients exhibited complications such as infections, periprosthetic fractures, or prosthetic fractures as of most recent follow-up.

**Conclusion:**

CCFE with an INCS represents a viable approach to massive femoral diaphyseal defect with short proximal femur repair, as patients can achieve good functional outcomes and early weight-bearing with proper individualized rehabilitative interventions, all while exhibiting low rates of procedure-related complications.

## Introduction

The surgical removal of large femoral malignancies can yield short femoral metaphyseal juxta-articular segments that can be difficult to accurately reconstruct (1). Reconstructive approaches in these patients include total femur replacement (TFR) (2), the use of inactivated autologous bone grafts (3), osteoarticular allografts (4), or a combination of both autografts and allofrafts (5, 6). While TFR can obviate the need to amputate the affected limb and is associated with positive functional outcomes during the early stages of patient follow-up, this procedure is often associated with undesirable outcomes including infection, local recurrence, aseptic loosening, hip disarticulation, and limb-length discrepancies (2, 7, 8). The use of inactivated autologous bone grafts offers several advantages including lower operative costs, appropriate anatomical matching, physiological reconstruction, and the lack of any need for a bone bank, but this procedure is also subject to limitations including the potential for infection, internal fixation failure, nonunion, and fracture of the inactivated bone (3). While the use of osteoarticular allografts can support the physiological reconstruction of target defect sites while preserving host bone integrity (9), larger allografts are generally associated with an elevated risk of infection, delayed union, nonunion, or graft fracture (4). Combined autografts and allografts combine the biological activity of free vascularized fibular grafts (FVFGs) with the initial mechanical strength of allografts. The Capanna technique has been reported to lessen the impact of complication (graft fracture, and delayed union or nonunion) (10–13). However, the risk of anastomosis failure by thrombosis is a concern (14). In addition, previous study showed that there was little difference in the percentage of graft fractures when comparing allografts with and without this vascularized graft (15).

The surgical removal of distal femoral tumors with proximal metaphyseal extension can often lead to the incidence of massive femoral diaphyseal defects (MFDD) with a short proximal femur (SPF), and optimal approaches to treating these defects remain to be established. In contrast to other forms of reconstructive surgery, the literature pertaining to the use of large femoral endoprosthesis is somewhat limited. However, the use of customized femoral endoprostheses can obviate the requirement for prolonged immobilization following allograft- or autograft-based reconstructive procedures, offering a means of immediately improving stability while promoting rapid recovery, early weight-bearing, a shorter duration of hospitalization, and a more rapid return to daily life and postoperative neoadjuvant chemotherapy or radiotherapy treatment, as appropriate (16, 17). However, these endoprostheses are often associated with both mechanical and non-mechanical complications including infection, periprosthetic fracture, breakage of the implant, and aseptic loosening (18). Insufficient contact area is available between the endoprosthetic stem and the cancellous bone in cases of MFDD with an SPF, and the inadequacy of cancellous bone in the trochanteric region can impact cement interdigitation for the straight cemented stem.

In our institution, SPF is defined as a residual proximal femur of ≤110 mm in length when measured from the pyriform fossa to the level of the osteotomy. In these cases, the residual segment is likely to be insufficient to accept a standard 150 mm intramedullary cemented stem (19). In an effort to better match the residual proximal femur while increasing the surface area of contact between the cancellous bone and endoprosthetic stem, a customized cemented femoral endoprosthesis (CCFE) with an intra-neck curved stem (INCS) was thus utilized for reconstructive procedures in cases of MFDD with an SPF. This study is the first to our knowledge to report clinical outcomes associated with such a reconstructive approach. As such, this analysis was primarily developed with the aim of detailing preliminary clinical outcomes and other experiences associated with INCS-based reconstruction, with a particular focus on associated mechanical stability.

## Materials and methods

### Ethical considerations

This retrospective study received ethical approval from the institutional ethics committee, and all patients provided written informed consent.

### Patients

**Figure 1:**
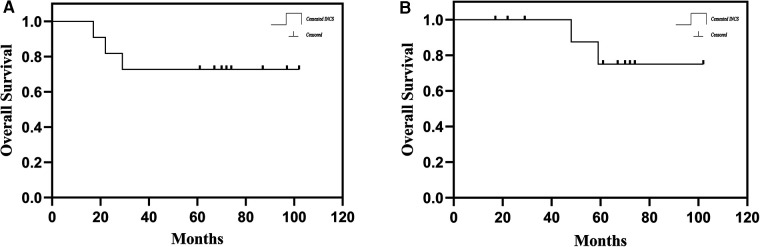
(**A**) the overall patient survival of hip-preserving reconstruction using a customized cemented femoral endoprosthesis with an INCS. (**B**) Survival to aseptic loosening of cemented INCS. (INCS: intra-neck curved stem).

**Figure 2:**
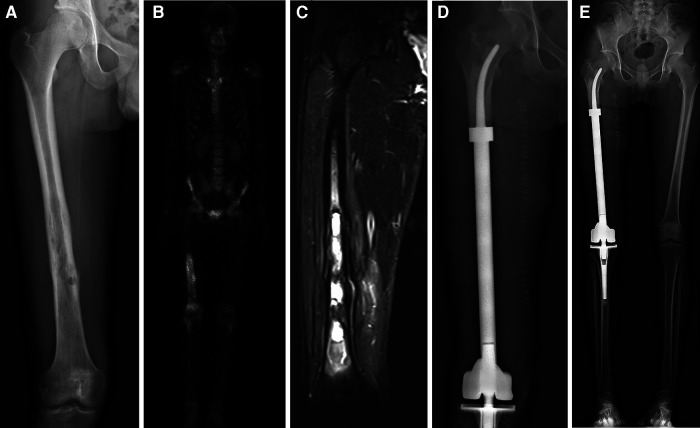
All patients underwent *en bloc* tumor resection followed by reconstruction with the customized cemented femoral endoprosthesis with an intra-neck curved stem. (**A**) Anteroposterior radiograph of the right femur of a patient with a distal femoral myofibroblastic sarcoma. (**B**) Single-photon emission whole-body Computed tomography image. (**C**) Magnetic resonance image of the patient's right upper leg. (**D**) Postoperative radiograph of the femur. (**E**) Full-length x-ray films of lower extremities in anteroposterior view at 7 days after surgery.

From 2013 to 2016, 11 total patients (6 male, 5 female; mean age: 26 years, range: 12–62 years) underwent distal femoral reconstruction procedures performed using a CCFE with an INCS. The average follow-up duration for these patients was 63 months (range: 17–102 months), during which three patients succumbed to lung metastases at 17, 22 and 29 months post-reconstruction, respectively (Figure 1A). Two patients developed aseptic loosening at 48 and 59 months post-reconstruction (Figure 1B). One patient developed local disease recurrence and ultimately succumbed to lung metastases. Surgical staging was performed as per the Enneking bone and soft tissue sarcoma staging system (20) (Table 1). Prior to definitive surgery, all patients underwent biopsy procedures. Preoperative x-ray, magnetic resonance imaging (MRI), computed tomography (CT), and single-photon emission CT approaches were used to establish the required length of bone to be resected (Figure 2). Patient demographic and clinical characteristics such as age, sex, tumor size, defect length, and residual proximal femur length were recorded.

**Table 1:** Surgical indications and stage of disease.

Patient no.	Age	Sex	Diagnosis	Metastasis	Enneking stage	Indication
1	13	F	Osteosarcoma	0	IIB	Primary sarcoma
2	21	M	Myofibroblastic sarcoma	0	IIB	Primary sarcoma
3	42	M	Osteosarcoma	Lung	IIIB	Primary sarcoma
4	62	M	Osteosarcoma	0	IIB	Primary sarcoma
5	29	F	Osteosarcoma	0	IIB	Primary sarcoma
6	23	F	Osteosarcoma	0	IIB	Primary sarcoma
7	12	M	Ewing sarcoma	0	IIB	Primary sarcoma
8	27	F	Ewing sarcoma	0	IIB	Primary sarcoma
9	18	F	Osteosarcoma	0	IIB	Primary sarcoma
10	16	M	Chondrosarcoma	0	IIB	Primary sarcoma
11	20	M	Ewing sarcoma	0	IIB	Primary sarcoma

M, male; F, female.

### Stem design and fabrication

After preoperative imaging-based determination of the tumor margins in each patient, the osteotomy plane was established. The stem was designed as an arc-shaped solid structure with a base that had a diameter that was 2–3 mm smaller than that of the inner surface of the inner femoral cortices. Stem curvature was designed in accordance with the medial cortex of the femoral neck. Strength was maintained by gradually reducing the stem diameter such that the diameter at the end of the curved stem region remained >10 mm. Our clinical team designed all stems, which were subsequently fabricated by Chunlizhengda Medical Instruments (Tong Zhou, Beijing, China).

### Surgical approach

One senior surgeon (Chongqi Tu) performed all procedures described in this study. All operations were conducted *via* a lateral approach with patients in the lateral recumbent position. Initially, the distal and medial segments of the femur were exposed. Tumors were then exposed, subjected to *en bloc* resection, and soft tissue was removed as appropriate based on preoperative simulations, with medullary tissue from the proximal femur being sent for frozen biopsy to confirm *en bloc* resection. Previous sites and needle biopsy tracks were additionally subjected to en block removal. Precise control of the osteotomy plane was maintained to minimize any risk of misfit between the residual proximal femur and the customized stem. Following tumor resection, the tip of a customized guide needle was inserted into the center of the femoral head using a mobile C-arm, after which a flexible reamer with different diameters and the customized guide needle were used to facilitate the gradual enlargement of the medullary cavity with the residual femur, ultimately producing a cone-shaped cavity. The prosthesis was then prepared to match the curvature of the residual femur and to correct for lower extremity alignment. A vacuum-mixing cement gun was used to inject bone cement into the medullary cavity, after which the curved stem was inserted into the residual proximal femur, with care being taken to ensure that no cement remained between the prosthesis and the soft tissue.

### Postoperative management

Following surgery, patients were routinely administered intravenous prophylactic antibiotics for 48 h. Rehabilitative programs were developed in an individualized manner based on the intraoperative assessment of each patient. In general, patients were subject to bed rest for 3–5 days, with their lower extremities being maintained in a neutral position with knee and ankle flexion and extension exercises being conducted in bed after 8 h. After 3 days, patients initiated hip flexion and abduction exercises, while after 7 days, patients began partial weight-bearing with the assistance of two crutches. After 21 days, patients began to progress to full weight-bearing.

During the initial 3 months after surgery, patients underwent monthly follow-up, followed by follow-up visits every 3 months for 2 years, with yearly visits thereafter. At each follow-up visit, a physical examination of the affected limb was conducted. A visual analog scale (VAS) was used to rate pain. Radiographic imaging of the reconstructed limb was conducted monthly during the first 3 months, every 3 months for the first year, every 6 months during the second year, and once per year thereafter. Lower limb function was assessed as per the Musculoskeletal Tumor Society (MSTS) scoring system (21). Postoperative stem positioning was assessed *via* x-ray. Femoral neck-shaft angles were measured before and after surgery. Procedure-related complications such as infection, periprosthetic fractures, aseptic loosening, and implant breakage were assessed.

### Statistical analysis

Differences between pre- and postoperative measurements were made *via* paired t-tests, with *p* < 0.05 as the threshold of significance. All data were analyzed using SPSS v 19.0 (IBM Corp, NY, USA).

## Results

### Radiographic analysis

**Figure 3:**
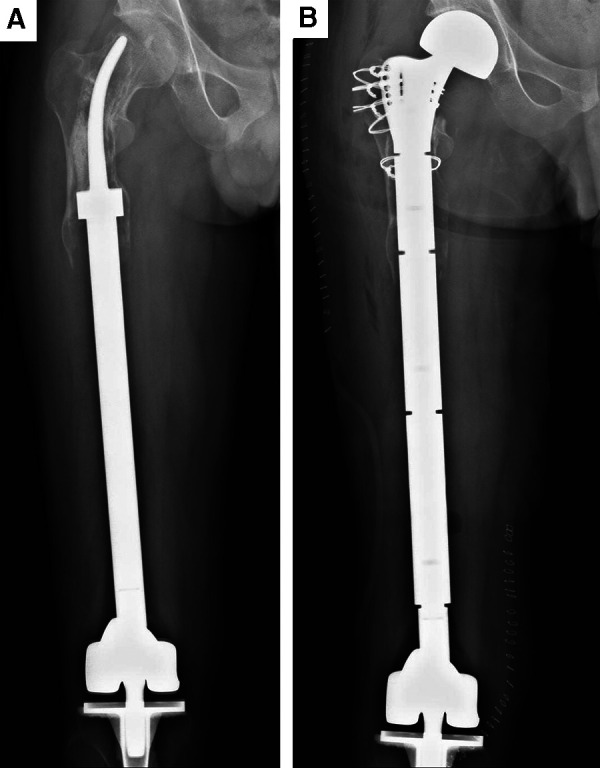
Aseptic loosening of the curved stem and revision surgery of case. (**A**) Aseptic loosening of the curved stem at the postoperative 97th month. (**B**) Aseptic loosening of the curved stem in case of revision surgery.

**Figure 4:**
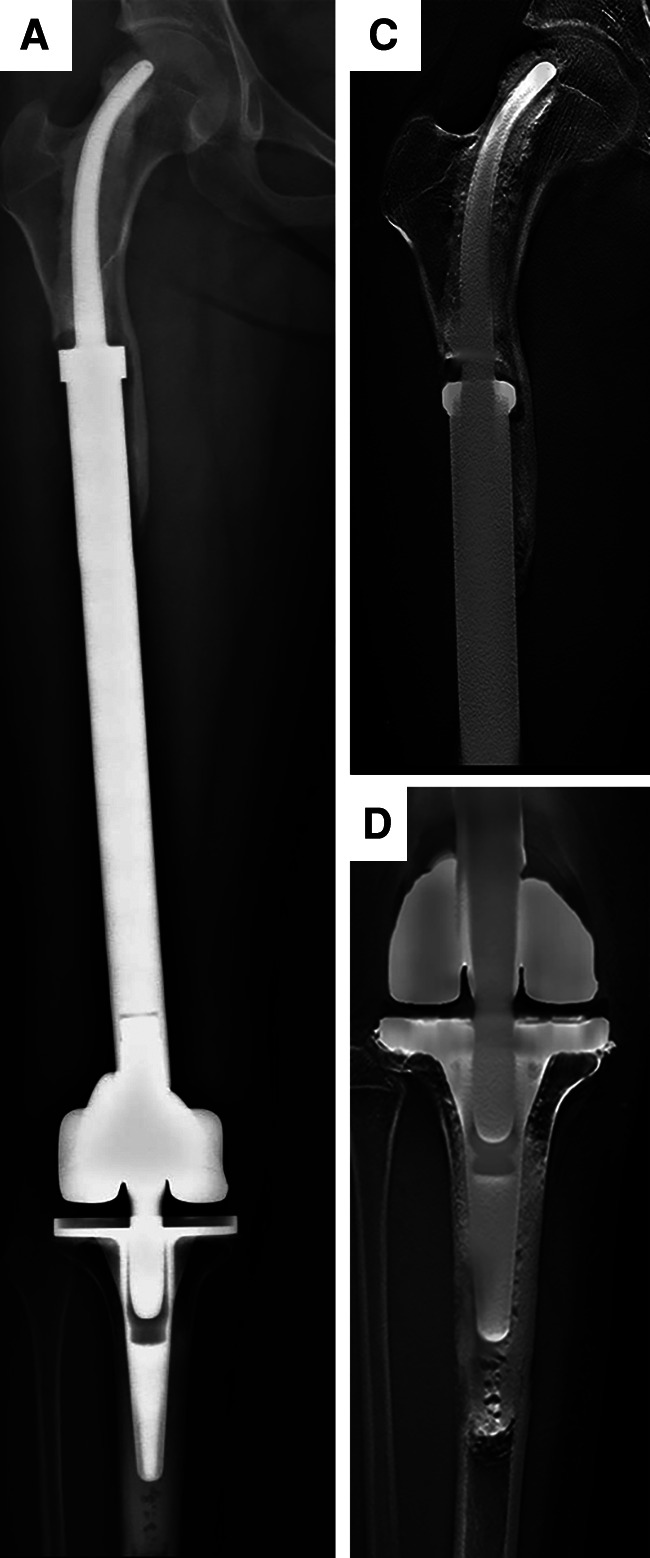
Radiographs showing the 102-month postoperative views of the customized cemented femoral endoprosthesis with an intra-neck curved stem placed during treatment for an osteosarcoma. (**A**) Posteroanterior radiograph of the entire femur. (**B**) Posteroanterior with Shimadzu Metal Artefact Reduction Technology (*T*-smart) views of the stem insertion region in the proximal femur. (**C**) Posteroanterior *T*-smart views of the stem insertion region in the proximal tibial.

x-ray and T-smart approaches were used to assess the bone-cement and cement-prosthesis interfaces in treated patients. In two cases, x-ray images revealed radiolucent lines at 48 and 59 months post-surgery, and these patients ultimately underwent revision surgical procedures and total femur replacement (Figure 3). No other patients exhibited such abnormalities. In one case, a patient underwent reconstructive surgery using a CCFE with an INCS following the resection of 82% of the femoral length in the context of massive tumor resection, and the most recent follow-up imaging of this patient revealed stable bone-cement and cement-prosthesis interfaces and neocortex formation (Figure 4). With the exception of two of the treated patients, lateral and posterior-anterior radiographic imaging revealed the stem tip to be located primarily in the middle third of the femoral head. No significant differences in pre- and postoperative femoral neck-shaft angle were evident in these patients (*p* = 0.410) (Table 2), consistent with acceptable INCS positioning.

**Table 2:** Details of the surgical technique and neck-shaft angle (preoperative/postoperative).

Patient no	Length of femur resection, mm	Percentage of femur resection length in the total femur length, %	Length of residual proximal femur, mm	Neck-shaft angle, ° (preoperative/postoperative)
1	340.50	81.67	76.40	129/132
2	337.60	75.78	107.90	122/123
3	332.30	80.69	79.50	130/126
4	349.00	80.79	83.00	130/125
5	333.20	77.00	99.50	124/127
6	321.50	77.34	94.20	128/123
7	315.90	77.83	93.60	130/126
8	348.20	81.93	76.80	128/133
9	314.10	75.52	101.80	131/125
10	367.00	80.45	89.20	129/134
11	352.30	78.85	94.50	135/129

### Functional analyses

**Figure 5:**
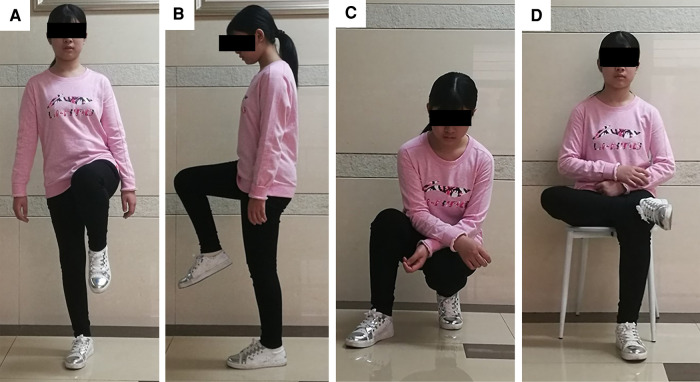
Limb function after INCS reconstruction at the postoperative 6th month of case. (**A**) This patient could stand up just with affected limb without any pain; (**B**) The knee and hip flexion of this patient was normal; (**C**) This patient could squat and stand up without any difficulty; (**D**) This patient could cross legs without any pain. (INCS: intra-neck curved stem).

The average MSTS score for this patient population as of most recent follow-up was 25 (range: 21–28). No surviving patients required crutches or other devices to aid walking at most recent follow-up. Two patients reported lower extremity pain when walking supported for over 3,000 m, with VAS scores of 3 and 2. Relative to preoperative VAS scores, patients exhibited significant overall reductions in pain (*p* < 0.0001) (Table 3). No other patients reported pain or Trendelenburg gait as of most recent follow-up (Figure 5).

**Table 3:** Results for patients undergoing distal femoral reconstruction with an intra-neck curved stem endoprosthesis.

Patient no	Oncological status	Follow-up (months)	Complication	VAS (preoperative/postoperative)	MSTS
1	NED	102	None	6/0	28
2	NED	97	Aseptic loosening	6/3	21
3	DOD	17	None	–	–
4	NED	87	Aseptic loosening	6/2	22
5	NED	72	None	5/0	25
6	DOD	22	Local recurrence	–	–
7	NED	70	None	5/0	27
8	DOD	29	None	–	–
9	NED	74	None	5/0	25
10	NED	61	None	5/0	25
11	NED	67	None	4/0	26

VAS, visual analog scale; NED, no evidence of disease; DOD, died of disease; MSTS, Musculoskeletal Tumor Society.

### Complications

One patient exhibited locally recurrent disease and ultimately succumbed to lung metastases at 22 months post-surgery. With the exception of the two patients that underwent revision surgery described above, there were no instances of aseptic loosening. None of the treated patients developed periprosthetic infections, periprosthetic fractures, neuropathy, implant breakage, or vascular incidents.

## Discussion

Optimal approaches to treating distal femoral tumors with proximal metaphyseal extension remain the subject of controversy. TFR is associated with both mechanical and non-mechanical complications (2). In addition, proximal femoral resection can also result in the opening of an additional compartment, and the hip joint may be affected in cases of local infection or recurrence, necessitating hemipelvectomy in certain cases to achieve the margins necessary to avoid further local recurrence (22) Hip joint preservation is critical to the improvement of lower limb function, and bone stock preservation is critical to permit future revision.

Reported hip-preserving reconstructive surgical approaches to date have included customized short medullary stems (23), stems with extra-cortical plates (19), allograft prosthetic composite (APC) (1, 24), stems with cross-fixation pins (16, 25) or the Compress® implant (26, 27). Short-stemmed endoprostheses have the potential to exhibit higher aseptic loosening and implant failure rates (28), with extracortical plates thus being employed in an effort to reduce these risks *via* supplemental fixation. APC preconstruction has been proposed as an alternative reconstructive approach for tibial and femoral sites (24, 29), offering advantages including endoprosthetic durability, intraoperative flexibility, and local bone stock availability. This approach, however, is subject to limitations such as infection, nonunion, and implant fracture incidence, and postoperative weight-bearing is generally delayed to permit the formation of an allograft-host junction (1). Stems with cross-fixation pins offer advantages including relatively low complication rates (30), but can be costly and necessitate time to facilitate the design and manufacturing process, making their use impractical for patients subject to time limitations associated with neoadjuvant chemotherapy treatment (24). Compressive osseointegration fixation can generate a stable, high-pressure bone-implant interface with the potential to avoid stress shielding (31, 32), but this approach is contraindicated when the cortical thickness at the bone-implant interface is <2.5 mm (33). This compression approach may thus be infeasible in children or patients that have undergone prior reconstructive procedures (24). Chemotherapy has also been shown to lower rates of bone-implant interface cortical hypertrophy, contributing to a trend towards decreased prosthetic survivorship in one report (34).

**Figure 6:**
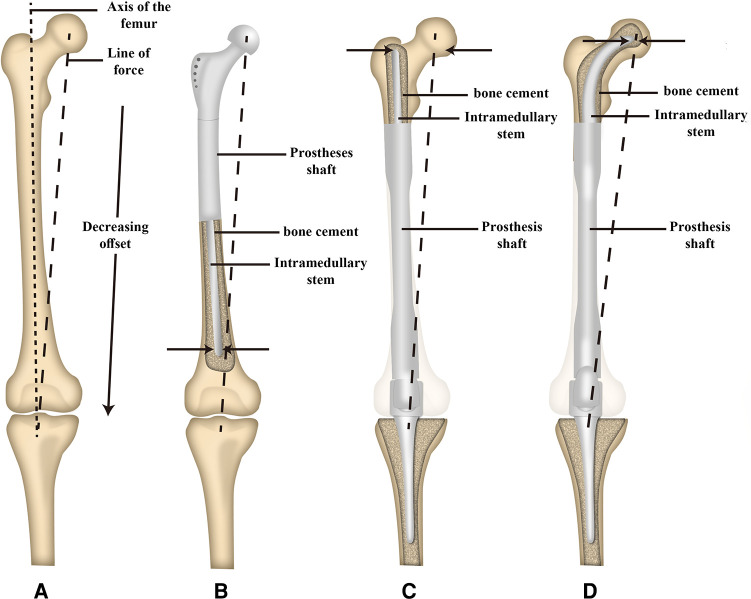
Schematic illustration of the offset distance between the line of force and the long axis of the femur and the offset distance of the tip of intramedullary stem between a proximal and distal femoral replacement. (**A**) The offset distance between the line of force and the long axis of the femur. (**B**) The offset distance of the tip of the intramedullary stem of proximal femoral replacement. (**C**) The offset distance of the tip of the intramedullary straight stem of distal femoral replacement. (**D**) The offset distance of the tip of the intramedullary curved stem of distal femoral replacement. [Adapted from ref. (26) with permission].

Promising short-stem endoprostheses available at present include the Compress® implant (26) and the Buxtehude stem (16). Rates of early aseptic loosening associated with the Compress® implant in prior studies range from 3.8%–14% (26, 35). The Buxtehude stem has been used in studies of patients with an SPF, exhibiting instances of fixation screw breakage and 12.5% early aseptic loosening incidence over the course of follow-up (16). In the present study, 2/11 patients developed aseptic loosening following femoral reconstruction, with this incidence rate being in line with rates reported previously for the Compress® implant and Buxtehude stem prostheses. Reasons for these rates of aseptic loosening may include the following: (1) The endoprostheses used for MFDD with an SPF reconstruction in the present study were designed to fit well with the proximal femur anatomy, with accurate INCS positioning being confirmed postoperatively; (2) Relative to a straight stem, the tip of the INCS yields a smaller offset distance such that the bending moment is smaller, potentially contributing to low rates of endoprosthesis loosening (36) (Figure 6). In addition, INCS make the force distribution of the residual proximal femur more even (Supplementary Figure S1); and (3) Achieving lasting fixation between bone and endoprostheses when using cemented straight femoral endoprostheses can be challenging owing to a lack of sufficient residual proximal femoral length. In cases of SPF, the proximal endpoint of the straight intramedullary stem is often present within the trochanteric region, which not only exhibits a large offset, but also does not contain sufficient cancellous bone (28). This lack of sufficient cancellous bone can adversely impact bone cement interdigitation, with the resultant distribution and thickness of this bone cement influencing the stability of the intramedullary endoprosthesis (37).

To enable greater intraoperative proximal femur retention, the tip of the stem was pressed to the femoral head-neck junction in two patients. While these patients exhibited good lower extremity function at early postoperative time points, they ultimately developed aseptic loosening of the prosthesis. We believe the reasons as follow: (1) Relative to the center of the femoral head, the stem tip will exhibit a increased distance of offset when located at the femoral head-neck junction; and (2) Relative to the center of the femoral head, the stem tip was subject to greater stress in the medial femoral head-neck junction region. In later procedures, the tip of the curved stem was pressed into the center of the femoral head when feasible during this reconstructive procedure. The positioning of the stem tip was deemed acceptable when located in the middle third of the femoral head in posterior-anterior and lateral radiographic views. Intramedullary stem stability can also be impacted by bone cement distribution and thickness (38). Lee et al. (39) reported a 2–5 mm mantle to be sufficient for bone cement penetration, with increasing thickness representing an effective means of reducing associated stress. For the present study in an effort to improve INCS stability, bone cement thickness at the stem base was increased slightly to 3–4 mm.

The average MSTS score among surviving patients in the present study (25 points) is in line with values reported in other prior studies (16, 26, 40). While complete lower extremity functional rehabilitation was not achieved, these patients did experience substantial pain relief and the ability to retain sufficient limb function to permit self-care. In addition, this operative approach was associated with a relatively quick postoperative recovery and allows for early weight-bearing, both of which are beneficial to patients. Patients in this study did not report any postoperative limitations in lower limb function in daily life. This approach yielded these positive outcomes for several reasons: (1) native hip joint preservation can decrease the potential for surgical disruption, minimizing muscular damage and preventing the degeneration of the articular surface that has the potential to occur when using prosthetic joints or osteoarticular allografts (41), thus allowing for maximal lower extremity functional restoration; (2) both the stability of the utilized endoprostheses and natural bodyweight transmission were beneficial to restoring limb function; and (3) this rehabilitative program was conducive to early functional training, leading to better function of the lower extremities. Periprosthetic infections and fractures are complications that are often observed following distal femur or diaphysis reconstruction (25, 42, 43). As of most recent follow-up, however, none of the patients included in this study had developed either of these complications.

There are several limitations to this study. For one, this is a single-center description of the experiences associated with procedures performed by one surgeon. The sample size in this study was limited, and the follow-up period was relatively short, potentially leading to a failure to note any uncommon complications associated with this operative approach. Moreover, this was a retrospective study with a noncomparative design owing to the rarity of prosthetic reconstruction procedures for MFDD with an SPF following malignant tumor resection, limiting the power of these results. However, the authors believe that, despite these limitations, this study can be instructive to other surgeons and researchers.

## Conclusion

In summary, the present study described preliminary outcomes associated with the use of a CCFE with an INCS as an alternative surgical procedure for cases of MFDD with an SPF, providing support for the safety and feasibility of this operative approach. This strategy has the potential to avoid risks associated with proximal femur resection, including dislocation, Trendelenburg limp, and the opening of an additional oncological compartment, while also allowing patients to achieve early weight-bearing and good lower limb function, all while maintaining low rates of procedure-related complications.

## Data Availability

The original contributions presented in the study are included in the article/**Supplementary Material**, further inquiries can be directed to the corresponding author/s.
