# Supplementary figures and images for: Hip-preserving reconstruction using a customized cemented femoral endoprosthesis with a curved stem in patients with short proximal femur segments: Mid-term follow-up outcomes
Source: Front Surg. 2022 Sep 22;9:991168. doi: 10.3389/fsurg.2022.991168 (PMC9632978; doi:10.3389/fsurg.2022.991168)

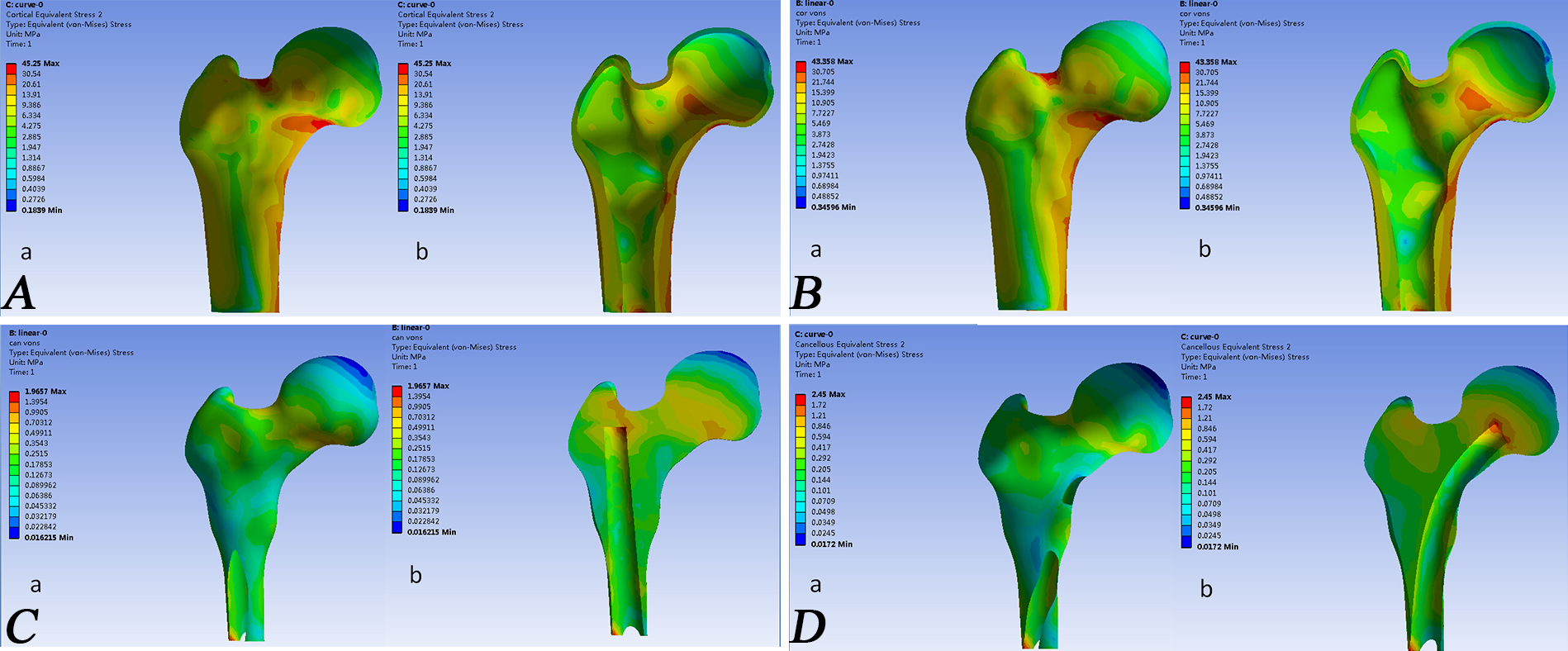

Supplement: Supplementary file 1 [file Image1.tif]
